# Connexin-36 distribution and layer-specific topography in the cat retina

**DOI:** 10.1007/s00429-019-01876-y

**Published:** 2019-06-06

**Authors:** Ildikó Telkes, Péter Kóbor, József Orbán, Tamás Kovács-Öller, Béla Völgyi, Péter Buzás

**Affiliations:** 1 Institute of Physiology, Medical School, University of Pécs, Szigeti út 12, Pécs, 7624 Hungary; 2 Szentágothai Research Centre, University of Pécs, Pécs, 7624 Hungary; 3 Department of Biophysics, Medical School, University of Pécs, Pécs, 7624 Hungary; 4 Department of Experimental Zoology and Neurobiology, University of Pécs, Pécs, 7624 Hungary; 5 Retinal Electrical Synapses Research Group, MTA-PTE NAP-2, University of Pécs, Pécs, 7624 Hungary; 6 Centre for Neuroscience, University of Pécs, Pécs, 7624 Hungary

**Keywords:** Calretinin, AII amacrine cell, Gap junction, Eccentricity

## Abstract

Connexin-36 (Cx36) is the major constituent of mammalian retinal gap junctions positioned in key signal pathways. Here, we examined the laminar and large-scale topographical distribution of Cx36 punctate immunolabels in the retina of the cat, a classical model of the mammalian visual system. Calretinin-immunoreactive (CaR-IR) cell populations served to outline the nuclear and plexiform layers and to stain specific neuronal populations. CaR-IR cells included horizontal cells in the outer retina, numerous amacrine cells, and scattered cells in the ganglion cell layer. Cx36-IR plaques were found among horizontal cell dendrites albeit without systematic colocalization of the two labels. Diffuse Cx36 immunoreactivity was found in the cytoplasm of AII amacrine cells, but no colocalization of Cx36 plaques was observed with either the perikarya or the long varicose dendrites of the CaR-IR non-AII amacrine cells. Cx36 puncta were seen throughout the entire inner plexiform layer showing their highest density in the ON sublamina. The densities of AII amacrine cell bodies and Cx36 plaques in the ON sublamina were strongly correlated across a wide range of eccentricities suggesting their anatomical association. However, the high number of plaques per AII cell suggests that a considerable fraction of Cx36 gap junctions in the ON sublamina is formed by other cell types than AII amacrine cells drawing attention to extensive but less studied electrically coupled networks.

**Electronic supplementary material:**

The online version of this article (10.1007/s00429-019-01876-y) contains supplementary material, which is available to authorized users.

## Introduction

Connexin-36 (Cx36) is the major constituent of mammalian retinal gap junctions positioned in key signal pathways (Bloomfield and Völgyi 2009; Völgyi et al. 2013). It has been shown that Cx36 gap junctions in the outer retina connect cones to decrease the signal-to-noise ratio, whereas heterologous Cx36 gap junctions between cones and rods underlie signaling through the so-called secondary rod pathway (DeVries et al. 2002; Schneeweis and Schnapf 1995; Tsukamoto et al. 1990; Deans et al. 2002; Schwartz 1975; Scholes 1975; Copenhagen and Owen 1976; Nelson 1977; Smith et al. 1986; Lee et al. 2003; Güldenagel et al. 2001; Feigenspan et al. 2001; O’Brien et al. 2004, 2012). Bipolar cell dendritic segments also form at least two distinct subpopulations of Cx36 gap junctions in the outer plexiform layer (OPL) including an aggregation of subpedicular gap junctions that likely connect various bipolar cell subtypes and also solitary Cx36 gap junctions that might connect nearby bipolar cells of the same subtype (Feigenspan et al. 2004; Kántor et al. 2017). In the inner retina, Cx36 gap junctions participate in a variety of connections (Bloomfield and Völgyi 2009; Völgyi et al. 2013), including bipolar-to-bipolar cell gap junctions located on overlapping bipolar cell axon terminals, amacrine-to-amacrine cell gap junctions, ganglion-to-ganglion cell gap junctions, ganglion-to-amacrine cell gap junctions, and, undoubtedly, the best-studied AII amacrine-to-ON-cone bipolar cell gap junctions (Güldenagel et al. 2001; Mills et al. 2001). The connection between AII amacrine cells and ON-cone bipolar cells is essential for scotopic vision as it maintains signaling through the primary rod pathway (Güldenagel et al. 2001; Deans et al. 2002) and it is likely common in most mammalian species (Kovács-Öller et al. 2017), although only a handful of animal models have been studied extensively in this regard.

Cats have been widely used as model animals in anatomical and physiological studies of the visual system (e.g., Bishop et al. 1962; Hubel and Wiesel 1962; Kolb and Famiglietti 1974; Blake 1979; Wässle et al. 1981a, b; Buzás et al. 2013; Kóbor et al. 2017); however, the focus of morphological investigations turned towards other popular animal models in the past decades including primates, rabbits, and mice. Therefore, there is a general paucity of information regarding the morphological substrates of electrophysiological results in the cat retina including the presence, distribution, and cellular specificity of Cx36 gap junctions. Moreover, many retinal neurons display eccentricity-driven morphological and density changes, and thus, it is expected that the topography of gap junction forming Cx36 plaques follows density changes of their expressing neurons. However, eccentricity-related changes in Cx36 distribution have not been examined in any species before. Therefore, we studied the distribution of Cx36 punctate immunoreactions in flat-mounted cat retina, and described both the layer-specific distribution and eccentricity-related changes of Cx36 plaque density. Moreover, we utilized CaR as a marker to stain cat AII cell somata and correlate their eccentricity-dependent density changes (Vaney 1985) with those of Cx36 plaques in the ON sublamina of the inner plexiform layer.

## Materials and methods

### Animals and sample preparation

Retinas of 3 adult cats (*Felis silvestris catus,* 1 male and 2 females, aged 0.83, 2.42 and 2.75 years) were used. The animals were kept and the experiments performed in accordance with Hungarian and European legislation. All procedures were approved by the Directorate for Food Chain Safety and Animal Health of the Baranya County Government Office, Hungary. Cats were overdosed with 5% isoflurane followed by injection of T61 (embutramide 250 mg/kg, tetracaine HCl 6.25 mg/kg, mebezonium iodide 63 mg/kg, Intervet, Boxmeer, The Netherlands) following unrelated physiological experiments and perfused intracardially with 4% paraformaldehyde in PBS (0.1 M phosphate-buffered saline, pH 7.5). The eyes were cut along the ora serrata and the vitreous body was removed. The posterior eyecups were postfixed overnight at + 4 °C and then transferred into cold PBS, the choroid carefully removed from the retinas, which were finally cut into upper, lower, nasal, and temporal quadrants using the optic disk as the center.

### Immunohistochemistry and confocal microscopy

Free-floating retinal quadrants were first incubated with blocking solution composed of 10% normal goat serum in antibody diluting solution (0.25% bovine serum albumin, 0.001% sodium azide, and 0.2% Triton X-100 in 0.1 M PBS) for 2 days. The same antibody diluting solution was used for all further antibodies. Incubation continued with a cocktail of the following primary antibodies: polyclonal anti-CaR produced in rabbit (1:2000 dilution, AB-5054 Merck Hungary, Budapest, Hungary) and monoclonal anti-Cx36 produced in mouse (1:1000 dilution, MAB3045, Merck Hungary, Budapest, Hungary) at + 4 °C for 4 days. The following incubations were done at + 4 °C overnight. Cx36 immunoreactivity was intensified using biotinylated anti-mouse IgG (H + L) (1:100 dilution, BA-2001, Vector Laboratories, Burlingame, CA, USA), and visualized with streptavidin-Alexa Fluor 488 conjugate (1:200 dilution in 0.1 M PBS, S32354, Invitrogen, Waltham, MA, USA). CaR immunoreactivity was visualized with goat anti-rabbit antibody coupled to Texas Red fluorophore (1:100 dilution, 111-075-003 Jackson ImmunoResearch Laboratories, West Grove, PA, USA). We washed the retinal pieces between the incubations five times for 10 min in 0.1 M PBS. Retinal pieces were mounted in Aqua-PolyMount (Polysciences, Warrington, PA, USA) medium with photoreceptor side up onto glass slides.

We inspected the flat-mounted retinas using a Zeiss LSM 710 confocal laser scanning microscope (Carl Zeiss, Jena, Germany) through a Plan-Apochromat 63 × objective lens (NA, 1.4). We took confocal stacks at selected regions of interest (ROIs, see below); the horizontal size of the ROIs was 135 × 135 μm and the stacks spanned depth from the outer nuclear layer to the optic fibers. Voxel size was 0.132 × 0.132 × 0.381 μm^3^.

### Measurement of retinal eccentricity and feature density

ROIs were selected randomly in each retinal quadrant (seven in the temporal, eight in the nasal, seven in the superior, and eight in the inferior quadrant) to cover all eccentricities. The location of each ROI was measured in polar coordinates with the optic disk as the center (range of distances from the optic disk 0.16–14.2 mm; mean ± SD 8.14 ± 3.74 mm). The eccentricities of ROIs were statistically not different in the four quadrants (Kruskal–Wallis test, *p* > 0.05). These coordinates were then converted into Cartesian coordinates of the entire flattened retina taking advantage of the fact that neighboring quadrants had common edges. Eccentricity of each ROI was calculated from the position 3 mm lateral from the optic disk; this position is in good agreement with the location of the area centralis in cat retina (Bishop et al. 1962; Hughes 1975).

Confocal stacks were further analyzed using the Fiji distribution of the ImageJ software package (Schindelin et al. 2012). Three types of CaR-immunoreactive cell bodies (1204 cells altogether) were differentiated in the inner retina by visual inspection of their CaR and Cx36 staining intensities and laminar position (see in the Results). Their positions were marked in each stack using the Cell Counter plugin and their number was divided by the area of the ROI to obtain areal density in mm^−2^.

The areal density of Cx36 plaques was determined using automated particle counting on preprocessed images as follows. From each stack, a single optical section was selected at 70% of the depth of the inner plexiform layer, i.e., in the ON sublamina. This depth corresponded to the maximum of Cx36 immunoreactivity, as shown in Fig. 4g. The selected optical section was high-pass filtered using the Bandpass Filter tool (pass band between 1 and 50 pixels) to remove large-scale variations of pixel intensity, e.g., due to unevenly scattered light. The result was then converted to binary image using automatic threshold in the Threshold tool (default IsoData method). This resulted in an image where Cx36 plaques were represented by isolated, roughly elliptical black dots. We also applied the Watershed tool to split concave shapes that could have resulted from overlapping dots. In some ROIs, a mask was applied to exclude regions out of focus or minor tissue damage. We identified and measured the area of each dot using the Analyze Particles tool. Dots with an area < 0.13 μm^2^ (about three pixels) were excluded to reduce the effect of pixel noise. The number of remaining dots was divided by the area of the ROI (minus the area of the mask if it was applied) to obtain areal density in mm^−2^.

To test whether the density data are significantly different in the four quadrants, we compared the densities of each of the three CaR-positive cell types of interest and Cx36 plaques using ANOVA as well as the Kruskal–Wallis test and found no significant differences (*p* > 0.05) between the quadrants for either of the parameters tested. Therefore, density measurements from the quadrants were pooled for the further analysis.

### Classification of labeled amacrine cells in the inner plexiform layer by cluster analysis

For cell density measurements, we classified amacrine cells by visual inspection as described above. To test whether this classification is supported by more objective criteria, we performed cluster analysis of a sample of 182 stained amacrine cells (16% of the total) as follows. We selected five stacks from the total of 30 for this procedure (two in the nasal quadrant and one in each of the other quadrants; eccentricities 2.8 mm, 4.4 mm, 8.1 mm, 11.3 mm, and 13.9 mm). Using ImageJ software, we drew regions of interest around each neuron that we had identified by visual inspection in the INL, irrespective of which cell type they had been assigned to previously. The ROIs were always drawn into the optical section where the cell was in sharpest focus and showed its widest profile. We then measured (using the Measure tool of ImageJ) the mean pixel intensities on both imaging channels (Cx36 and CaR) within each of the ROIs. We converted the measured values into *Z*-scores to express them in units of standard deviations relative to the mean. Since we calculated *Z*-scores for each stack separately, differences in mean intensity and contrast between the stacks were largely eliminated. Finally, we used *k*-means clustering [kmeans function in Matlab (MathWorks, Natick, MA, USA)] to classify the ROIs into two clusters. After this procedure, we compared the assignment of cells to the clusters and the cell types identified by visual inspection. As shown in Table 1 (and by the marker types in Fig. 3), the two methods of classification provided identical results in all but 2 of the 182 analyzed cells.

**Table 1:** Number of cells in the INL classified by visual inspection or cluster analysis

Cell type by visual inspection	Cell type by cluster analysis		
	Cluster 1	Cluster 2	Total
Strongly CaR + regular amacrine cell	31	1	32
AII amacrine cell	1	149	150
Total	32	150	182

The data show that, for a sample of 182 cells, the two methods of classification provided identical results in all but two cases

### Data analysis

ANOVA and Kruskal–Wallis test were done in IBM SPSS (IBM Corporation, Armonk, NY, USA), significance level was set to *p* < 0.05. *K*-means clustering and Pearson’s correlation analysis of density and eccentricity data were done using Matlab. Correlation was accepted as significant if *p* < 0.05. Model curve fitting was done using the Solver tool in Excel (Microsoft, Redmond, WA, USA).

## Results

The main interest of the present study was to describe and analyze the laminar and tangential distributions of connexin-36 expression in relationship to cell types expressing the Ca^2+^-buffer protein calretinin (CaR) in the cat retina. CaR is a marker of several retinal neuron types in various mammalian species including the cat (Völgyi et al. 1997; Kovács-Öller et al. 2017; Goebel and Pourcho 1997; Pasteels et al. 1990). In particular, CaR is expressed by cat horizontal cells and AII amacrine cells. Here, we utilize this information as a guideline for further investigations.

### Calretinin-immunoreactive cell types of the cat retina

**Fig. 1:**
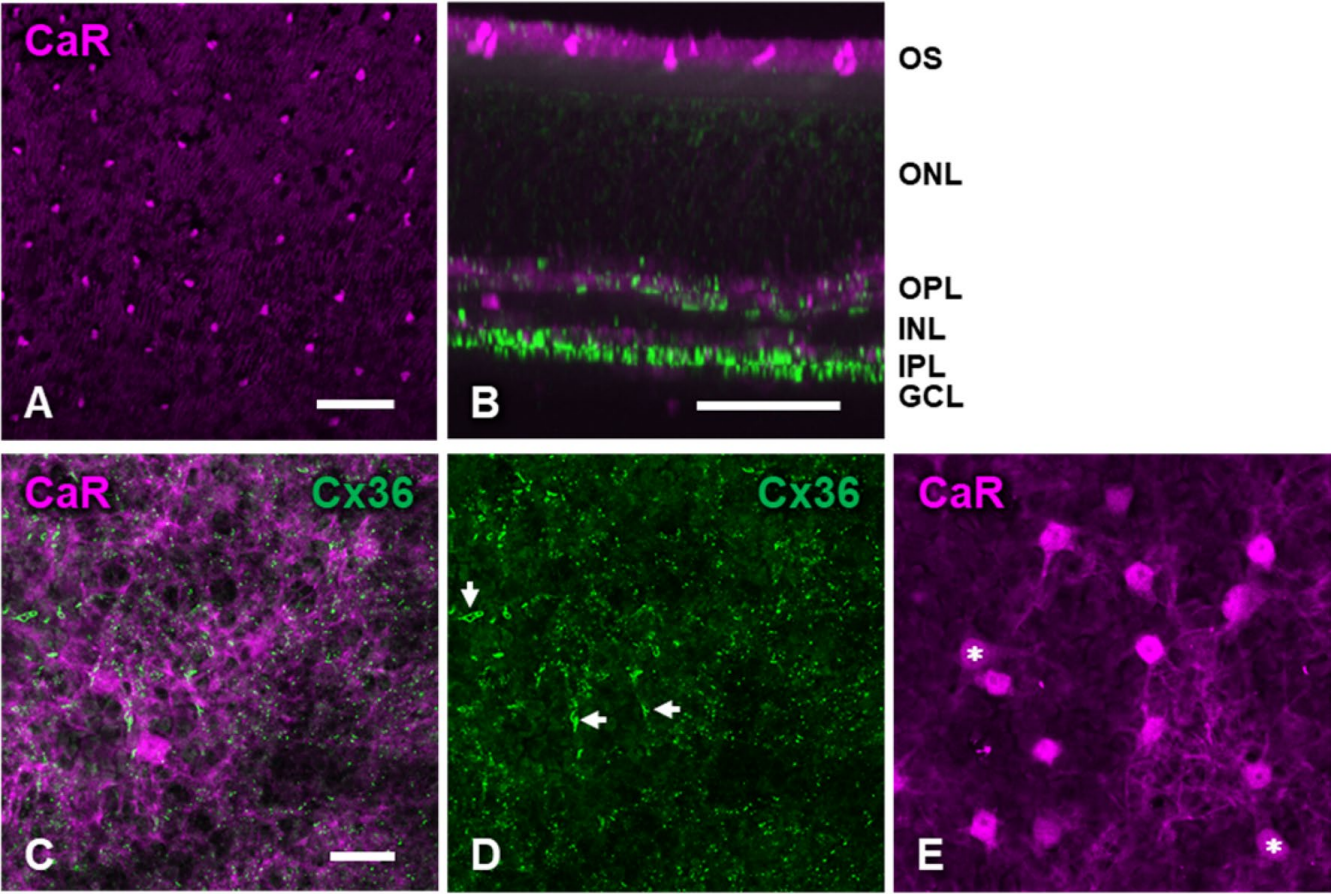
Immunohistochemical labeling of calretinin (magenta) and connexin-36 (green) in the outer layers of the cat retina. **a** Cone photoreceptor outer segments revealed by the anti-CaR antibody in the flat-mounted retina. Eccentricity, 13.9 mm. **b** Side view reconstruction of the same confocal stack as in **a** showing all retinal layers. **c** Composite image of both markers with focus on the outer plexiform layer. Connexin-36 plaques were not systematically associated with horizontal cells dendrites. Eccentricity, 4.4 mm. **d** Same frame as A showing Cx36-positive plaques and occasional elongated structures (arrows). **e** Cell bodies and proximal dendrites of CaR-positive horizontal cells. Same field of view as A but focus is slightly deeper on the top of the inner nuclear layer. Asterisks indicate type-A horizontal cells with thicker but more weakly labeled proximal dendrites. Scale bar, 20 μm

Our results confirmed earlier findings that CaR is present in several cell types of the cat retina (Pasteels et al. 1990; Goebel and Pourcho 1997; Jeon and Jeon 1998; Kovács-Öller et al. 2017). In the outer layers, cone photoreceptor outer segments (Fig. 1a, b) and horizontal cells (Fig. 1c, e) contained CaR. The outer plexiform layer (OPL) was clearly delineated by the dense network of labeled horizontal cell dendrites (Fig. 1b, c). As seen in Fig. 1e, some weakly labeled CaR-positive horizontal cells had thicker proximal dendrites and are, therefore, likely type-A cells. The more numerous, prominently labeled cells with relatively thinner dendrites correspond to type-B horizontal cells consistent with earlier descriptions (Boycott et al. 1978; Pasteels et al. 1990; Jeon and Jeon 1998).

**Fig. 2:**
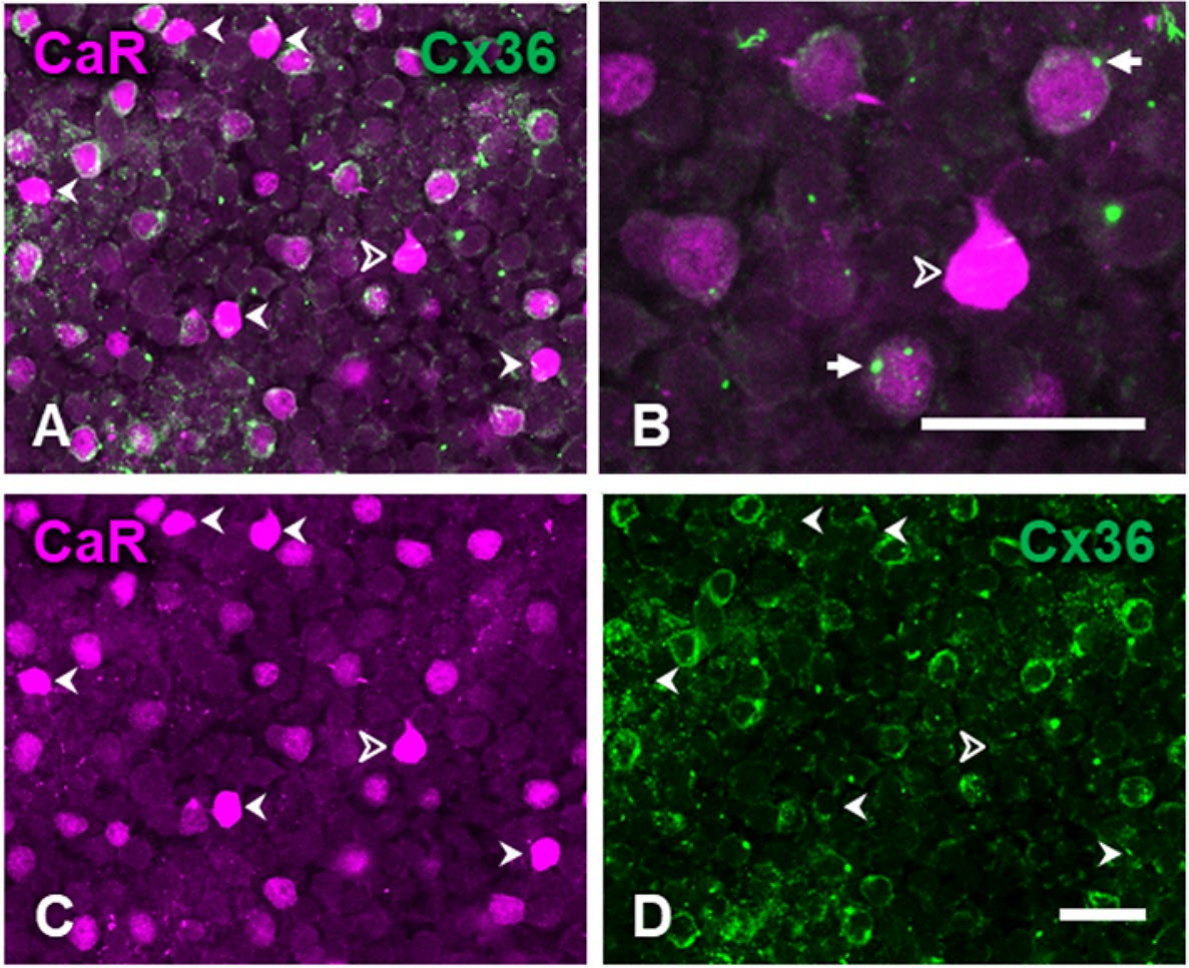
Calretinin-immunoreactive amacrine cells and connexin-36 immunoreactivity of the inner nuclear layer. Eccentricity, 4.4 mm. **a** Composite image of calretinin (magenta) and connexin-36 (green) double label. Arrowheads indicate strongly CaR-positive amacrine cells. AII cells show weaker CaR-labeling and diffuse cytoplasmic Cx36 expression. **b** Close-up of the strongly CaR-positive amacrine cell (indicated by the open arrowhead in panels **a** and **c**) surrounded by several AII amacrine cells. Large Cx36 plaques are often seen associated with double labeled AII cells (arrows). **c**, **d** Show CaR and Cx36 immunolabeling, respectively, for the same region as in **a**. Note the lack of Cx36 label at the locations of strongly CaR-positive cell bodies (arrowheads in **d**). Scale bar, 20 μm

Further CaR-immunoreactive cell bodies were identified as amacrine cells based on the location of their somata in the proximal inner nuclear layer (INL). Two sets of CaR-immunoreactive amacrine cell bodies could be differentiated; one with strong CaR label in the entire cell body and a more numerous population with relative weak CaR label (Fig. 2a, b). The strongly CaR-labeled amacrine cells were often seen to extend CaR-IR varicose processes into the IPL (Figs. 2b, 5a). The weakly CaR-immunoreactive amacrines were the only neurons in our material that contained diffuse Cx36 immunoreactivity in their cytoplasm suggesting strong and continuous expression of this protein (Fig. 2a, d). The processes of these weakly CaR stained cells were not labeled, suggesting that CaR was mainly expressed in the soma. However, based on earlier descriptions in the cat (Pasteels et al. 1990; Goebel and Pourcho 1997; Jeon and Jeon 1998; Kovács-Öller et al. 2017) as well as other species (Pasteels et al. 1990; Wässle et al. 1995; Völgyi et al. 1997), these cells are most likely AII amacrine cells, key constituents of the rod signaling pathway (Kolb and Nelson 1983; Bloomfield and Völgyi 2009; Deans et al. 2002; Bloomfield and Völgyi 2004; Völgyi et al. 2013).

**Fig. 3:**
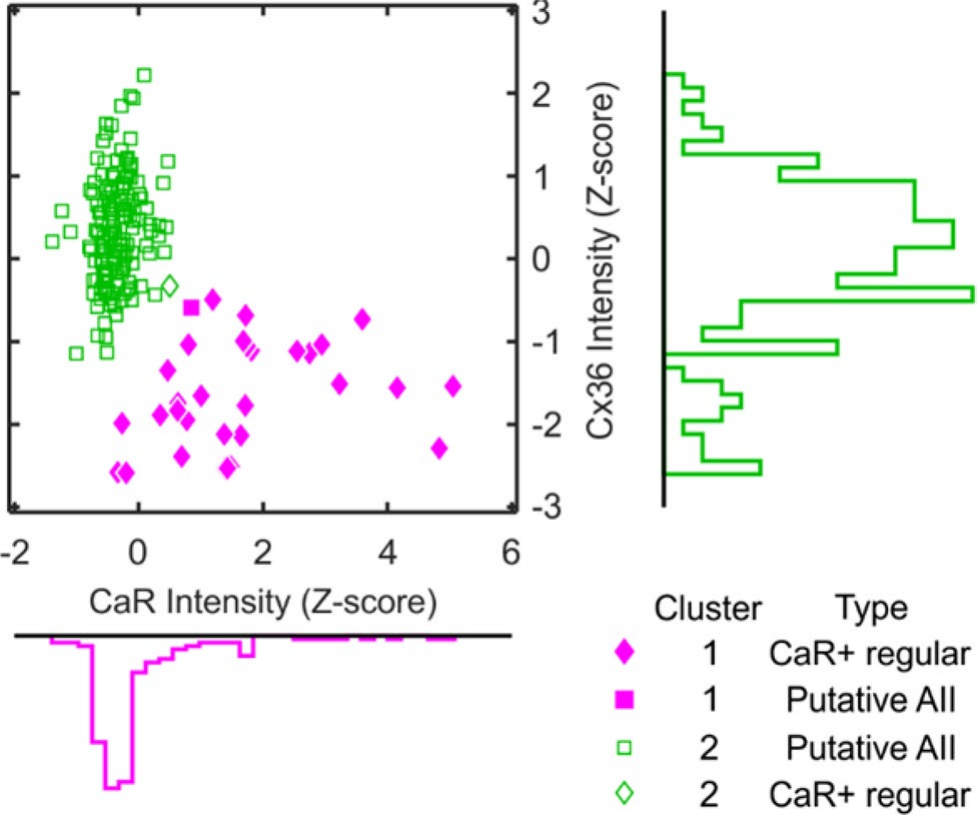
Classification of amacrine cells in the inner plexiform layer using cluster analysis based on CaR and Cx36 staining intensity. Scatter plot shows the labeling intensity of a sample of cell profiles (*n* = 182) on the imaging channels for CaR (abscissa) and Cx36 (ordinate) in units of standard deviations around the mean. Filled and open markers indicate the two clusters defined by *k*-means clustering, respectively. Marker shape indicates the two cell types established by visual inspection. Marginal histograms show the distributions of staining intensities

The distinction of two amacrine cell types on the basis of CaR and Cx36 labeling intensity is further supported by our analysis illustrated in Fig. 3. Staining intensities of a sample of 182 amacrine cells were measured for CaR and Cx36, and the two intensities (expressed as *Z*-scores, see “Methods”) were used to divide the sample into two clusters. The more numerous and densely packed cluster of cell profiles was characterized by weaker CaR label and stronger Cx36 label. The second cluster represented cells with weaker Cx36 label and stronger CaR label. These two groups corresponded nearly perfectly to the AII and strongly CaR-positive amacrine cell classes described by morphological criteria above (see Table 1 and marker types in Fig. 3).

**Fig. 4:**
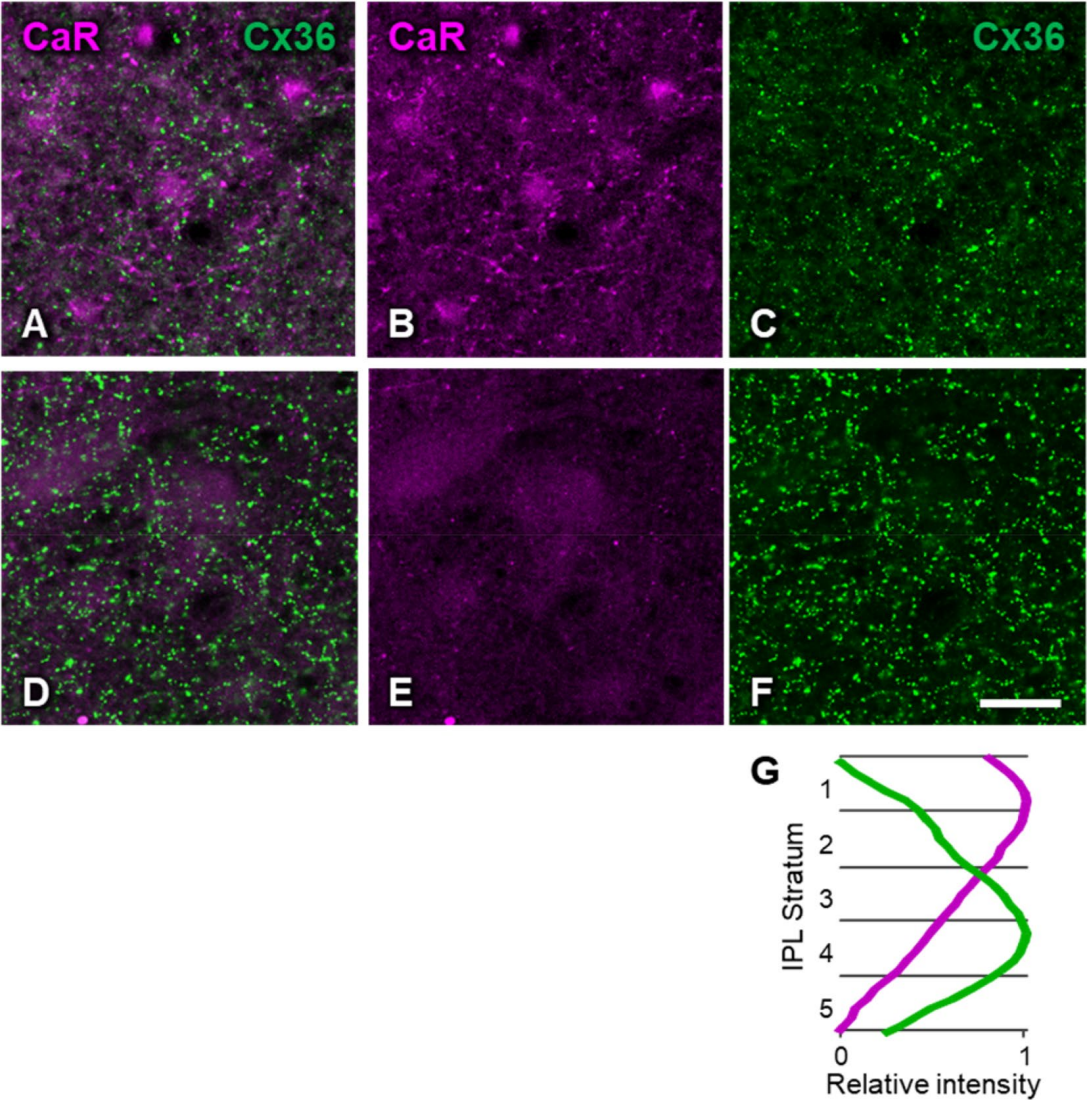
Horizontal view of the inner plexiform layer double labeled for calretinin and connexin-36. **a**–**c** Focus on stratum 2 of the IPL where calretinin label had the highest density. **d**–**f** Focus on stratum 4 of the IPL where connexin-36 label had the highest density. **a**, **d** Composite image. **b**, **e** CaR only. **c**, **f**, Cx36 only. Scale bar, 20 μm. Eccentricity, 2.8 mm. **g** Fluorescence intensity profiles along the *Z*-axis of the confocal stack shown in **a–f** for CaR (magenta) and Cx36 (green). The ordinate shows depth within the IPL with the five strata indicated. Fluorescence intensity is scaled between 0 and 1 within the IPL. The differential staining of the IPL sublaminae can also be seen in Fig. 1b

**Fig. 5:**
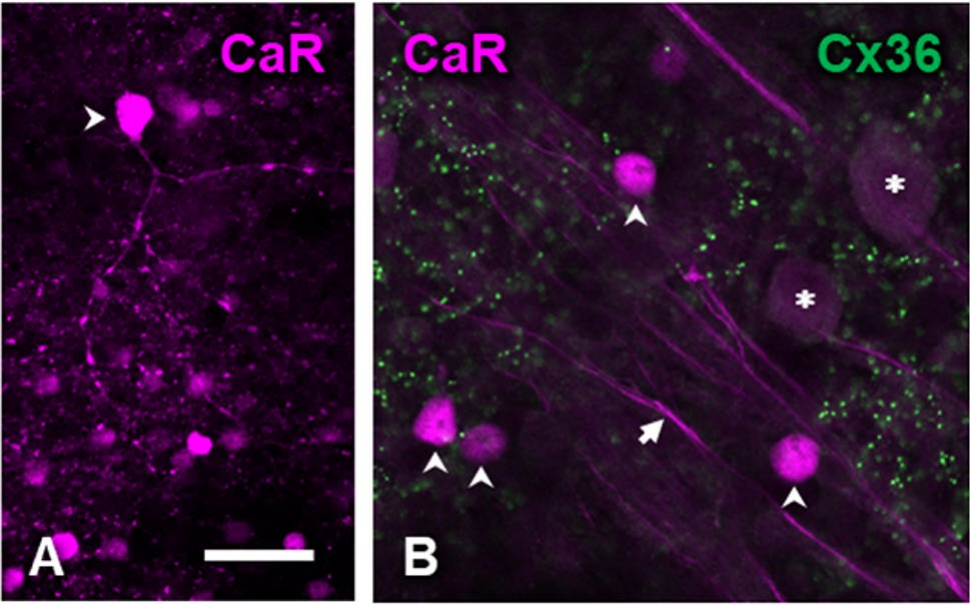
**a** Strongly CaR-positive (magenta) regular amacrine cells were the main source of the dense meshwork of varicose dendrites in the OFF sublamina of the IPL (also see Fig. 4a, b). **b** In the ganglion cell layer, CaR was expressed by small cell bodies (arrowheads) and larger, weakly labeled ganglion cells (asterisks). CaR-immunoreactive optic fibers (arrow) and connexin-36 plaques (green) from the inner plexiform layer are also seen. Eccentricity **a** 10.6 mm; **b** 11.3 mm. Scale bar, 20 μm

In the inner plexiform layer, we found the highest density of CaR-positive dendrites in the OFF sublamina (Figs. 1b, 4b, g). Well-stained CaR-positive dendrites with en-passant boutons could often be followed back to the strongly CaR-positive regular amacrine cells or to CaR-positive cell bodies in the ganglion cell layer (Fig. 5a, b). In the ON sublamina, CaR label was found in varicose dendrites similar to those in the OFF sublamina, but their density was much lower (Fig. 4e, g).

The presence of immunoreactive optic fibers suggested that some ganglion cells contained CaR, as well. Although we did not see labeled axons leaving them, potential candidates are the lightly labeled cell bodies of the ganglion cell layer with diameters over 10 μm (Fig. 5b). In the ganglion cell layer, stronger CaR expression was found in small cell bodies (Fig. 5b) which also lacked labeled axons. With their soma diameters (Feret diameter) of 9.98 ± 2.31 μm (mean ± SD, *n* = 28) were they on the boundary between displaced amacrine cells and the smallest known ganglion cells (Isayama et al. 2000; Boycott and Wässle 1974; Wässle et al. 1987) and they were also somewhat larger (two-sample *t* test for unequal variances *p* = 0.028) than the strongly CaR-positive regular amacrines in our sample (Feret diameter 8.79 ± 1.64 μm, *n* = 32) (Fig. 5). Therefore, we refer to them as “strongly CaR + cells of the GCL” for the purpose of the present paper.

### Connexin-36 plaques and their relationship to calretinin-immunoreactive structures of the cat retina

In both plexiform layers, the Cx36 antibody labeled small puncta (Figs. 1, 4) showing the typical appearance and localization of immunolabeled gap junctions seen in earlier studies (Güldenagel et al. 2000, 2001; Feigenspan et al. 2001; Mills et al. 2001; Deans et al. 2002). In the OPL (Fig. 1), we observed small Cx36 puncta in irregular clusters or sometimes strings without consistent association with the CaR-labeled horizontal cell dendrites (Fig. 1a, c, also see Supplementary Material). The pattern of these Cx36 puncta did not seem to correspond to the rather regular patterns of cone pedicles or rod spherules; therefore, they very likely belonged to the subpedicular conglomerate or the solitary Cx36 gap junctions maintained by bipolar cell dendritic processes (Kántor et al. 2017). The Cx36 immunolabel also revealed occasional, elongated structures (Fig. 1c, arrows). Although reminiscent of large connexin-50-positive gap junction plaques described in rabbit A-type horizontal cells (O’Brien et al. 2006), these structures only showed minimal overlap with the CaR-labeled horizontal cells (see Supplementary Material).

In the inner plexiform layer, the density of Cx36 immunoreactive puncta varied with depth. In the OFF sublamina, their density was generally lower (Fig. 4a, c). Interestingly however, large plaques were found at the level of the INL/IPL border. Many of these large plaques were associated with the weakly stained amacrine cell bodies (Fig. 2a, b). However, the highest density of Cx36 plaques was found in the ON sublamina of the IPL, which also showed the least CaR-positive structures (Figs. 1b, 4d–f). A *Z*-axis profile through the image stack of Fig. 4g illustrates the clear distinction between the intensities of CaR and connexin labels within the IPL. No Cx36 plaques were found to be associated with cell bodies of the ganglion cell layer (Fig. 5b).

### Eccentricity-dependent variations in the density of calretinin-positive amacrine cells

The laminar patterns of CaR and Cx36 expression described above corresponded to the findings by Kovács-Öller and colleagues (Kovács-Öller et al. 2017) who studied cross sections of mid-peripheral cat retina. Here, we extended these investigations and examined eccentricity-dependent changes in the inner retina where Cx36 is thought to be mainly expressed by AII amacrine cells. We selected 30 regions of interest in the retinal flat mounts and measured the densities of the three CaR-immunoreactive amacrine cell types described above as well as the density of Cx36 puncta. Based on our initial qualitative observations, the presence of CaR-IR cell types and the laminar pattern of Cx36 puncta did not appear different in various retinal regions. Our statistical analysis, however, revealed that the densities of certain structures were clearly eccentricity-dependent, whereas others were independent of retinal eccentricity.

**Fig. 6:**
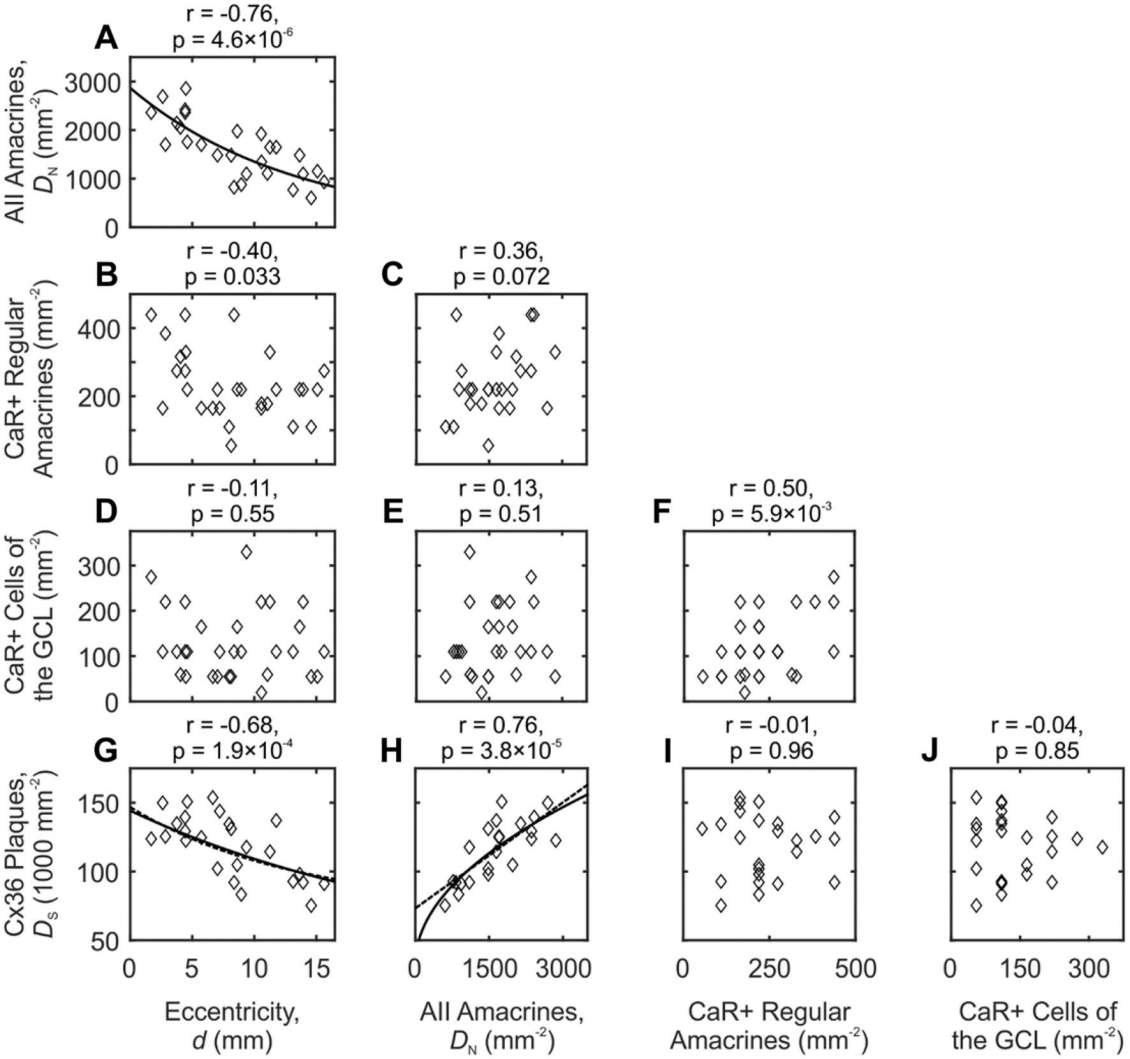
Pairwise comparisons of the densities of the three CaR-IR amacrine cell types innervating the inner plexiform layer, Cx36 puncta, and eccentricity. Each data point (diamonds) corresponds to one region of interest. Pearson’s correlation coefficients (*r*) and their significance levels are shown for each pair of measures; *p* < 0.05 was regarded as significant. Alignment of data points in some panels is due to the small (integer) number of strongly labeled amacrine cells in a region of interest. Curves in **a**, **g,** and **h** show best-fit models of the data. **a** Exponential decay (*R*^2^ = 0.60). **h** Dashed line, linear model (*R*^2^ = 0.58); solid line, square root model (*R*^2^ = 0.62). Curves in **g** were obtained by substituting the respective models in **h** into the equation of the curve in **a** to estimate the eccentricity dependence of Cx36 density (*R*^2^ = 0.49 and *R*^2^ = 0.50, respectively)

Figure 6a, b, and d shows the relation between distance from the area centralis and density values of the CaR-immunoreactive cell types of the inner plexiform and ganglion cell layers. The strongest correlation with eccentricity was apparent for AII amacrine cells (Fig. 6a, Pearson’s correlation coefficient *r* = − 0.76, *p* = 4.6 × 10^−6^), whereas weaker correlation was found for the strongly CaR-positive regular amacrine cells (Fig. 6b, *r* = − 0.40, *p* = 0.033). Both correlations were negative, which is characteristic for the cone photoreceptor density and for components of cone-associated pathways in the cat retina (Goodchild et al. 1996; Steinberg et al. 1973; Hughes 1975). A good approximation of AII neuron density ($D_{\text{N}}$) as a function of eccentricity (*d*) could be given by an exponential relationship in the form of $D_{\text{N}} = D_{{{\text{N}}0}} {\text{e}}^{ - d/\lambda }$, where *D*_N0_ is the AII cell density in the area centralis and *λ* is the space constant of the exponential decay. Best-fit values (*R*^2^ = 0.60) for this model obtained by least-squares minimization were $D_{\text{N0}}$ = 2866 mm^−2^ and *λ* = 13.29 mm; the corresponding curve is shown in Fig. 6a. The estimated density in the area centralis is, thus, lower than the value measured by Vaney (1985); this discrepancy can be the result of under-sampling the central region or it may result from the difference in the method of cell labeling.

The density of the strongly CaR-positive cells of the GCL was, on the other hand, not correlated with eccentricity (Fig. 6d) suggesting that these cells are less involved in functional channels concerned with cone pathways and spatial resolution of vision. Interestingly, their density was, nevertheless, positively correlated with the strongly CaR-positive regular amacrine population (Fig. 6f, *r* = 0.50, *p* = 5.9 × 10^−3^). The two populations were, however, not symmetrical as there were about twice as many regular cells (median ratio of cell numbers in all regions of interest = 2.0, *n* = 21).

### Determinants of connexin-36 plaque density

The anti-CaR antibody labeled AII amacrine cells too weakly to allow unambiguous identification of their dendritic trees (also see Pasteels et al. (1990)), but current evidence from several mammalian species including the cat suggests that a major source of Cx36 immunoreactivity in the IPL is the gap junctions formed by AII amacrine cells with other AII cells or with ON-cone bipolar cells (Feigenspan et al. 2001; Kovács-Öller et al. 2017; Mills et al. 2001; Kántor et al. 2017). In the following, we describe the eccentricity-dependent change in Cx36 plaque density, and then, we attempt to explain this variation by considering AII amacrine cells and other cell types as sources of the labeled gap junctions.

We counted the Cx36 labeled puncta in each region of interest at the level of the ON sublamina, where the highest Cx36 labeling intensity was observed in the IPL (Fig. 4g). As shown in the bottom row of Fig. 6, Cx36 density (*D*_s_) was negatively correlated with eccentricity (Fig. 6g, *r* = − 0.68, *p* = 1.9 × 10^−4^). Furthermore, Cx36 density was positively correlated with the density of AII amacrine cells (Fig. 6h, *r* = 0.76, *p* = 3.8 × 10^−5^), which is consistent with their anatomical association. No such correlation was seen with the densities of the strongly CaR-positive cell types (Fig. 6i, j).

**Fig. 7:**
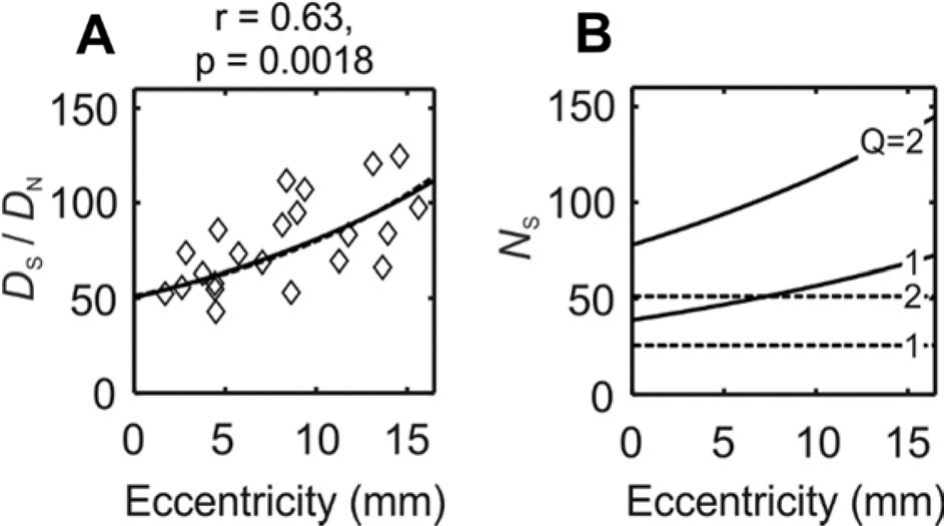
**a** The density ratio of Cx36 puncta and AII amacrine cells as a function of eccentricity for each region of interest (diamonds). Curves are derived as the ratio of the respective linear (dashed line) or square root models (solid line) of Fig. 6g and a. Note that an estimate of gap junctions per AII cell must take into account the type of connections that these gap junctions are involved in (see text). **b** Estimates of the numbers of gap junctions per AII amacrine cell as a function of eccentricity. Curves are obtained from the linear (dashed lines) or square root models (solid lines) assuming either only homocellular (*Q* = 2) or only heterocellular (*Q* = 1) gap junctions

Can the observed density of Cx36 puncta be explained by assuming AII cells alone as their source? The answer to this question depends on our estimate of the number of gap junctions formed by a single AII amacrine cell. If (for the sake of argument) all Cx36 puncta were located on the dendrites of AII amacrine cells, the ratio of their densities ($D_{\text{S}} /D_{\text{N}}$) would provide a measure of gap junctions formed by a single AII cell. In our data set, this ratio varied between 42.9 and 124.7 (median = 73.5) and increased with eccentricity (Fig. 7a). However, it is important to note that the interpretation of these values depends on the cell types connected through the labeled gap junctions.

Three types of connections can be distinguished; (1) homocellular gap junctions between labeled AII cells, (2) heterocellular gap junctions between labeled AII cells and unlabeled synaptic partners (likely ON-cone bipolar cells); and (3) gap junctions between unlabeled cells. If all synaptic partners were other labeled AII cells, then each Cx36 plaque would signify a gap junction for two AII cells, so that the number of gap junctions per cell (*N*_S_) would be given by $2D_{\text{S}} /D_{\text{N}}$. If, on the other hand, all synaptic partners were unlabeled, the number of gap junctions formed by a single AII cell would be $D_{\text{S}} /D_{\text{N}}$. If all Cx36 puncta belonged to AII cells, the above multiplier (we symbolize it with *Q*) would be somewhere between 1 and 2, because AII cells formed both types of connections. If, however, Cx36-positive gap junctions were also formed between non-AII neurons, the multiplier *Q* would decrease further, approaching 0 as the proportion of unidentified synaptic partners increases.

As long as the ratio of the three types of connections is unknown, we can only give an upper estimate of *N*_S_ using *Q* = 2, i.e., assuming purely homocellular gap junctions. When the model curves of Fig. 7a are accepted as reasonable estimate of the $D_{\text{S}} /D_{\text{N}}$ density ratio, this could mean that *N*_S_ is between 100 and over 200 depending on eccentricity. Independent data against which this figure could be verified vary greatly depending on the method and species. From electron microscopic reconstructions of AII cell dendrites, it turns out that the number of gap junctions per AII amacrine dendrite is between 10 (cat, Sterling et al. (1988), mouse, Tsukamoto and Omi (2013)) and 31^1^ (rabbit, Marc et al. (2014)). Quite surprisingly, the highest estimates of gap junction contacts per AII cell come from light-microscopic work [over 100 in the rabbit (Mills et al. 2001), about 140–170 in the mouse (Meyer et al. 2014)]. In any case, these data suggest that a considerable number of Cx36 puncta derive from non-AII neurons.

Because of technical limitations, studies at the cellular or ultrastructural level give usually little insight about how synapse numbers change with eccentricity even if it is well established that the size of dendritic trees increases for many retinal cell types including AII amacrines (Vaney 1985). In the following, we will consider two simple models to approximate the relationship of $D_{\text{S}}$ vs. $D_{\text{N}}$, as shown in Fig. 6h. Common in both models is that they express Cx36 plaque density as the sum of two fractions. One fraction depends on AII cell density and it takes into account how the number of connections might scale with the size of the AII cell dendritic tree; this term accounts for the gap junctions formed by AII cells. The second term is a constant baseline density of Cx36 plaques ($D_{\text{SB}}$), which is independent of AII cell density (and, thus, eccentricity). Derivation of the model equations is given in the Appendix.

The first, linear model assumes that each AII amacrine contributes equal number of Cx36 plaques (*N*_S_). After adding the baseline plaque density, we obtain a simple linear equation (Eq. 3 of the Appendix):

$$D_{\text{S}} = \frac{{N_{\text{S}} }}{Q}D_{\text{N}} + D_{\text{SB}} .$$

Fitting the linear model to the data (dashed line in Fig. 6h) by least-squares minimization delivered the equation $D_{\text{S}} = 25.66 D_{\text{N}} + 73 265$. The slope of the regression line estimates $N_{\text{S}} /Q$; therefore, the estimated number of gap junctions per AII cell is between 26 and 51 for values of *Q* between 1 and 2 (Fig. 7b).

The linear model is, however, in conflict with examples from the literature, suggesting that the density of synapses per unit area of dendritic membrane is held constant within one class of retinal neuron at any eccentricity (Vardi et al. 1989; Kier et al. 1995). This scaling of synapse numbers is also plausible if the electrotonic impact of synaptic inputs is to be held constant in the face of increasing dendritic tree sizes towards the periphery. A model that incorporates this scenario would take the following simple form (Eq. 11 of the Appendix):

$$D_{\text{S}} = q\sqrt {D_{\text{N}} } + D_{\text{SB}} .$$

where *q* is constant. With optimized parameters (*q* = 2087 mm and *D*_SB_ = 32,540 plaques/mm^2^), this model resulted in slightly better fit to the data (solid line in Fig. 6h). According to this model, the estimated number of gap junctions on AII cells would be between 39 and 145 per cell depending on eccentricity and the value of *Q* (Fig. 7b).

Three points can be concluded from the above analysis. First, both mathematical models gave similar density estimates for the range of eccentricities available. Although the curves capture the trend of the data, the relatively high residuals suggest that further factors, for example regional differences, also influence the density of Cx36 plaques. Notwithstanding this fact, a considerable fraction of Cx36 plaques (estimated by the baseline densities) was not explained by anatomical association with AII amacrine cells. Finally, the estimated number of gap junctions per AII amacrine cell was equal or greater than the numbers known from the literature (Sterling et al. 1988; Mills et al. 2001; Tsukamoto and Omi 2013; Marc et al. 2014; Meyer et al. 2014). We consider the implications of this discrepancy in the last section of the Discussion.

## Discussion

We analyzed the laminar and tangential distributions of punctate connexin-36 immunoreactive structures, the presumed light-microscopic correlates of the most prevalent type of retinal gap junctions, and their relationship to calretinin-immunoreactive structures in whole mounts of cat retina. The main result of the study was that the density of Cx36 plaques decreased with eccentricity and this decrease correlated with the eccentricity-dependent density fall-off of type AII amacrine cells, while it was not correlated with the densities of CaR-positive non-AII amacrine cells. The density of Cx36 plaques was, however, several times higher than expected if AII amacrine cells were their sole source.

### Comparison of laminar distribution of connexin-36 with other species

Cx36 has been found in both synaptic layers of the retinas of all mammalian species studied so far (Kovács-Öller et al. 2017; Güldenagel et al. 2000). The outer plexiform layer contains gap junctions responsible for two of the best-known functional syncytia in the retina. Of these, cone terminals are coupled through channels made up of Cx36 (Deans et al. 2002; Feigenspan et al. 2001; Mills et al. 2001), whereas the horizontal cell syncytium features other connexin types including connexin-50 and connexin-57 [mouse, Janssen-Bienhold et al. (2009); Palacios-Prado et al. (2009); (Pan et al. 2012), rabbit, Huang et al. (2005); O’Brien et al. (2006); Cha et al. (2012), and carp, Greb et al. (2017)]. Interestingly, the pattern of punctate Cx36 labels in the cat OPL does not seem to be compatible with either of these (also see Kovács-Öller et al. (2017)), so that Cx36 gap junctions in the cat outer retina likely belong to bipolar cells and could be localized in the subpedicular conglomerate (Feigenspan et al. 2004; Kántor et al. 2016; Bolte et al. 2016) or on their more proximal dendrites (Kántor et al. 2017). It has been suggested that cone bipolar cells form Cx36 gap junctions based on the observation that the cone dominant retinas of humans or guinea pigs show more robust Cx36 expression in the outer retina than cats, dogs, or ferrets, where the ratio of rods is high (Kovács-Öller et al. 2017). Since the various cone bipolar cell types are thought to be the starting elements of largely separated, functionally specific pathways, it would be important to see which bipolar cell types are involved in electrical synaptic coupling and what functional roles these connections may support.

The occasional elongated Cx36-positive structures which we observed in the OPL may bring to one’s mind the large gap junction plaques formed by connexin-50 between type-A horizontal cell dendrites in the rabbit (O’Brien et al. 2006) or axon terminals of mouse type-B horizontal cells (Dorgau et al. 2015). Although horizontal cell gap junctions have been shown to stain in the carp (Liu et al. 2009) with the same Cx36 antiserum that we used, no similar report is known to us about mammalian horizontal cells. Cross reaction of the Cx36 antibody with connexin-50 in the cat is a possibility, although the same monoclonal primary serum has been used by several research laboratories without reporting similar problems (Pereda et al. 2003; Han and Massey 2005; Ivanova et al. 2015; O’Brien et al. 2012). It is important to note that it is unlikely that our quantitative analysis of the IPL is affected, because connexin-50 or 57 is not expressed in that layer (Völgyi et al. 2013).

The density peak in the ON sublamina of the inner plexiform layer is the most conservative feature of the laminar distribution of Cx36 in mammalian retinae (Kovács-Öller et al. 2017); the cat retina is no exception in this respect (Fig. 4g). It is likely that a large (although not yet quantified) proportion of Cx36 expression in the IPL belongs to two sites of connection in the primary rod pathway that communicates rod signals towards ganglion cells, namely homotypic junctions between AII amacrine cells and the (probably heterotypic) gap junctions between AII amacrine cells and ON-cone bipolar cells. The predominance of Cx36 in the ON sublamina of the IPL is in line with what is known about the subcellular localization of these connections on the AII amacrine cell dendrite (Dacheux and Raviola 1986; Strettoi et al. 1992; Chun et al. 1993; Mills and Massey 1995). An interesting morphological feature of the AII–AII network seen in an electron microscopic study of the cat retina (Vardi and Smith 1996) is that large gap junctions are formed between AII cell bodies, sometimes by virtue of long, thin appendages of the cells. The prominent, solitary Cx36 plaques seen on many AII cell bodies in our material (Fig. 2) are likely the light-microscopic correlates of these gap junctions.

Besides gap junctions that interconnect neurons, there is a possibility that a subpopulation of the Cx36 plaques seen in the light microscope in fact represents hemichannels. It has been shown that Cx36 forms functional hemichannels in pancreatic islet β-cells (Pizarro-Delgado et al. 2014) and neuronal cell cultures (Schock et al. 2008). Although we cannot rule out the possibility, there is no evidence yet for Cx36 forming functional hemichannels in the living nervous tissue.

### Calretinin immunoreactivity and its colocalization with connexin-36

The Ca^2+^-buffer protein calretinin is a marker of distinct retinal cell types in various mammalian species including the rabbit (Völgyi et al. 1997; Massey and Mills 1999), monkey (Mills and Massey 1999), and human (Lee et al. 2016; Kántor et al. 2016). Our results confirmed earlier descriptions that, in the cat, anti-CaR antibodies label cone outer segments, horizontal cells, certain amacrine cells including the AII type and some ganglion cells (Pasteels et al. 1990; Goebel and Pourcho 1997; Jeon and Jeon 1998; Kovács-Öller et al. 2017).

The distinction of three CaR-IR cell types that innervate the IPL was important for our present study. Two of them were strongly labeled; those in the inner nuclear layer probably corresponded to the amacrine cells distinguished by Goebel and Pourcho (1997) on the basis of heavy CaR-labeling throughout their cell bodies. The sparser population of strongly CaR-positive cell bodies seen in the GCL may be heterogeneous that likely includes displaced amacrine cells as judged by their soma size and the lack of labeled axons (Fig. 5). They also resembled the “asymmetric CaR-positive cells” described by Pasteels et al. (1990) in labeling intensity, soma size, and areal density.

Our criterion for distinguishing AII amacrine cells, i.e., the colocalization of CaR and Cx36 in their cell bodies, was in line with the previous studies. Although the cytoplasmic Cx36 label has not been described before, it is not surprising given the importance of gap junction coupling in AII cell function (Bloomfield and Völgyi 2004) and the high turnover of connexin proteins (Wang et al. 2015). The relatively weaker CaR-labeling of AII amacrine cells (also observed by Goebel and Pourcho (1997) in cross-sectioned tissue) is probably the reason why the dendrites of AII cells did not show up in whole mounts. Furthermore, none of the other CaR-positive structures showed systematic colocalization with Cx36 (also see (Kovács-Öller et al. 2017)).

The density and tangential distribution of retinal cell types is a useful indicator of their functional roles. Measured densities of AII cells vary with the method of their identification in feline retina. Vaney (1985) used vital staining of whole retinas by DAPI and found a gradient from 5300 mm^−2^ in area centralis to 500 mm^−2^ in the periphery. Figures obtained from one-by-one identification of all cells of the outer nuclear layer in serial semithin sections (Macneil et al. 2009) or electron microscopic reconstructions (Sterling et al. 1988) were up to twice as high. Our method delivered densities between these results (Fig. 6a) with somewhat lower estimates in central retina and higher estimates in peripheral locations than Vaney (1985). Nevertheless, it is well established that AII cells are the most numerous type of amacrine cell in the cat retina (Macneil et al. 2009) consistent with the adaptation to rod-mediated vision of this animal. It is, therefore, notable that the centrally peaking AII amacrine cell density (Fig. 6a) (Vaney 1985; Macneil et al. 2009) resembles the distribution of cones and their associated pathways rather than the distribution of rods (Goodchild et al. 1996; Steinberg et al. 1973; Hughes 1975) or rod bipolar cells (Greferath et al. 1990; Macneil et al. 2009). This suggests that AII cells improve the spatial resolution of rod vision in central retina by taking advantage of the high resolution of cone pathways (Macneil et al. 2009; Wässle et al. 1995; Strettoi et al. 2018).

### Topography of connexin-36 expression in the retina

The present study is, to our knowledge, the first attempt to describe and analyze the large-scale tangential distribution of gap junctions in the retina of any species. Our observation, a general decrease in the density of connexin-36 plaques with eccentricity, may not seem surprising given that the densities of many cell types including AII amacrine cells follow a similar trend and Cx36 is the constituent of their gap junctions. However, these data need to be in line with a realistic model of how the contribution of AII cells changes towards the periphery. We found that the total contingent of Cx36 plaques can be well described as the sum of two fractions; one that is constant and independent from the density of AII amacrine cells (the “baseline” density) and another that is in some predictable relationship with it.

Our data are compatible with a realistic scenario of how the number of electrical synapses scales with the increasing size of the dendritic trees towards the retinal periphery (the “square root model”, see Appendix). However, this increase could only account for the AII-dependent fraction of Cx36 plaques if several times more gap junctions are assumed per AII cell than known from the electron microscopic literature (Sterling et al. 1988; Tsukamoto and Omi 2013; Marc et al. 2014). Our estimates can be better reconciled with figures from recent studies using fluorescent labeling of Cx36 plaques (Mills et al. 2001; Meyer et al. 2014), which suggest up to ten times more gap junction contacts in a single AII cell. Moreover, gap junctions could also be located on cell types that were not labeled in our material but whose retinal distribution is correlated with that of the AII cells. In this case, the AII-dependent fraction of the Cx36 population could, in fact, include gap junctions connecting non-AII cells. Similarly, the baseline fraction of Cx36 plaques is best explained by gap junctions located on non-AII neurons whose collective distribution is rather flat across the retina.

Our analysis, therefore, calls one’s attention to extensive networks coupled through Cx36 connexons that appear to exist besides the much studied AII amacrine-cone bipolar cell network in the inner retina. These functional syncytia are expected to involve at least as many connections as the AII network. It has been shown that ganglion cells of the mammalian retina form homologous ganglion-to-ganglion and heterologous ganglion-to-amacrine cell gap junctions (Xin and Bloomfield 1997) and many of these connections are comprised by Cx36 (Schubert et al. 2005; Pan et al. 2010; Völgyi et al. 2009). Most amacrine cell subtypes also form an inner retinal network of their own (Xin and Bloomfield 1997). Finally, the axon terminals of both ON- and OFF-cone bipolar cells establish inner retinal electrical synapses with neighboring bipolar cells. These connections might form a conduit for signaling between various subtypes of bipolar cells (Dacey et al. 2000; Marc et al. 1988; Mills 1999; Luo et al. 1999; Kántor et al. 2017).

### Electronic supplementary material

Below is the link to the electronic supplementary material.

Supplementary material 1 (PDF 668 kb)

## Appendix: Derivation of the link between cell density and synapse density based on rules of dendritic organization in the retina

We are interested in the relationship between the density of labeled neurons, *D*_N_, and the density of their labeled synapses, *D*_S_, both measured in a region of retina. We begin with estimating the number of synapses per neuron, *N*_S_. If the labeled cells do not make connections with each other through the labeled synapses, then $N_{\text{S}} = D_{\text{S}} /D_{\text{N}}$. If, on the other hand, all labeled synapses make connections between two labeled cells, then $N_{\text{S}} = 2D_{\text{S}} /D_{\text{N}}$. If some of the synapses are common and the rest is not, we have to use a multiplier that is between 1 and 2. We assume that this number *Q* is constant for a given cell type in our sample. Thus

1 $$N_{\text{S}} = Q\frac{{D_{\text{S}} }}{{D_{\text{N}} }}.$$

### Linear model

Here, we assume that the number of synapses, $N_{S}$, is the same for each individual cell. It follows from Eq. 1 that:

2 $$D_{\text{S}} = \frac{{N_{\text{S}} }}{Q}D_{\text{N}} .$$

Finally, we would also like to allow for a constant baseline density of synapses, $D_{\text{SB}}$, which is independent of cell density:

3 $$D_{\text{S}} = \frac{{N_{\text{S}} }}{Q}D_{\text{N}} + D_{\text{SB}} ,$$

which is the equation of a line with slope $\frac{{N_{\text{S}} }}{Q}$ and *y*-intercept $D_{\text{SB}}$.

### Square root model

Here, we will use two rules of dendritic organization described by Kier et al. (1995) for alpha and beta ganglion cells of the cat retina. The first rule observed by Kier and colleagues states that the density of synapses on a cell’s dendritic membrane is constant for any size of the dendritic field. A similar relationship has also been found for the varicosities of starburst amacrine cell dendrites (Vardi et al. 1989). The synapse density in the membrane ($D_{\text{SM}}$) can be written as follows:

4 $$D_{\text{SM}} = \frac{{N_{\text{S}} }}{{A_{\text{M}} }},$$

where $A_{\text{M}}$ is the total area of dendritic membrane. Kier and colleagues’ second rule says that the dendritic membrane area is proportional to the radius of the dendritic field ($r_{\text{F}}$). This relationship has also been observed recently for the dendritic trees of AII amacrine cells in the rat (Zandt et al. 2017). Therefore, we assume that:

5 $$A_{\text{M}} = ar_{\text{F}} ,$$

where *a* is constant. Although we could not measure the radii of the dendritic fields in our setting, we can deduce them from assuming constant coverage factor (*C*) (Cleland et al. 1975; Vaney 1985), which is defined as follows:

6 $$C = D_{\text{N}} A_{\text{F}} ,$$

where *A*_*F*_ is the area of the dendritic field given by: $A_{\text{F}} = \pi r_{\text{F}}^{2}$. Therefore

7 $$r_{\text{F}} = \sqrt {\frac{{A_{\text{F}} }}{\pi }} = \sqrt {\frac{C}{{\pi D_{\text{N}} }}} .$$

From Eqs. 4–7, it follows that

8 $$N_{\text{S}} = aD_{\text{SM}} \sqrt {\frac{C}{{\pi D_{\text{N}} }}} ,$$

meaning that the number of synapses per cell is inversely proportional to $\sqrt {D_{\text{N}} }$. Substituting this into Eq. 2 gives:

9 $$D_{\text{S}} = \frac{1}{Q}aD_{\text{SM}} \sqrt {\frac{C}{{\pi D_{\text{N}} }}} D_{\text{N}} = \frac{1}{Q}aD_{\text{SM}} \sqrt {\frac{C}{\pi }} \sqrt {D_{\text{N}} } .$$

We have assumed that everything left of $\sqrt {D_{\text{N}} }$ is constant, and therefore, $D_{\text{S}}$ is proportional to $\sqrt {D_{\text{N}} }$. Lumping the constants together in a factor *q* gives the following:

10 $$D_{\text{S}} = q\sqrt {D_{\text{N}} } .$$

Similar to the linear model, we would, finally, like to allow for some baseline synapse density that is independent of the density of the labeled neurons:

11 $$D_{\text{S}} = q\sqrt {D_{\text{N}} } + D_{\text{SB}} .$$

## Footnotes

1 The uniquely detailed reconstruction of a volume of rabbit IPL at the electron microscopic level by Marc et al. (2014) allows a good estimate. This study found 39 AII amacrine cells, 525 homocellular AII–AII gap junctions, and 172 heterocellular AII-cone bipolar cell gap junctions in the analyzed tissue volume. It follows from the data that the average number of homocellular gap junctions per cell is 2 × 525/39 ≈ 27, the average number of heterocellular gap junctions is 172/39 ≈ 4, and thus, 31 gap junctions are formed by one AII amacrine. We do not know, however, to what extent these observations can be applied to various regions of the cat retina.
