# Supplementary material for: Connexin-36 distribution and layer-specific topography in the cat retina
Source: Brain Struct Funct. 2019 Jun 6;224(6):2183–97. doi: 10.1007/s00429-019-01876-y (PMC6591202; doi:10.1007/s00429-019-01876-y)
Supplement: Supplementary file 1 — Supplementary material 1 (PDF 668 kb) [file 429_2019_1876_MOESM1_ESM.pdf]

SUPPLEMENTARY MATERIAL

Colocalization analysis of CaR and Cx36 in the outer plexiform layer

We performed the colocalization analysis in 4 stacks using the Fiji distribution of the ImageJ software package. From each stack, we chose 10 optical sections (3.8 µm) spanning the OPL. We separated the two imaging channels and then performed background subtraction (Process/Subtract Background) using kernel diameter of 100 pixels (13 µm). The Coloc2 plugin (Schindelin et al. 2017) was used to threshold the images using Costes' method and calculate Pearson's correlation coefficients for above-threshold pixels. The results are summarized in Supplementary Fig. 1. Pearson's correlation values for above-threshold pixels show weak negative correlation, which is evidence for the lack of colocalization or even anti-correlation between the two labels.

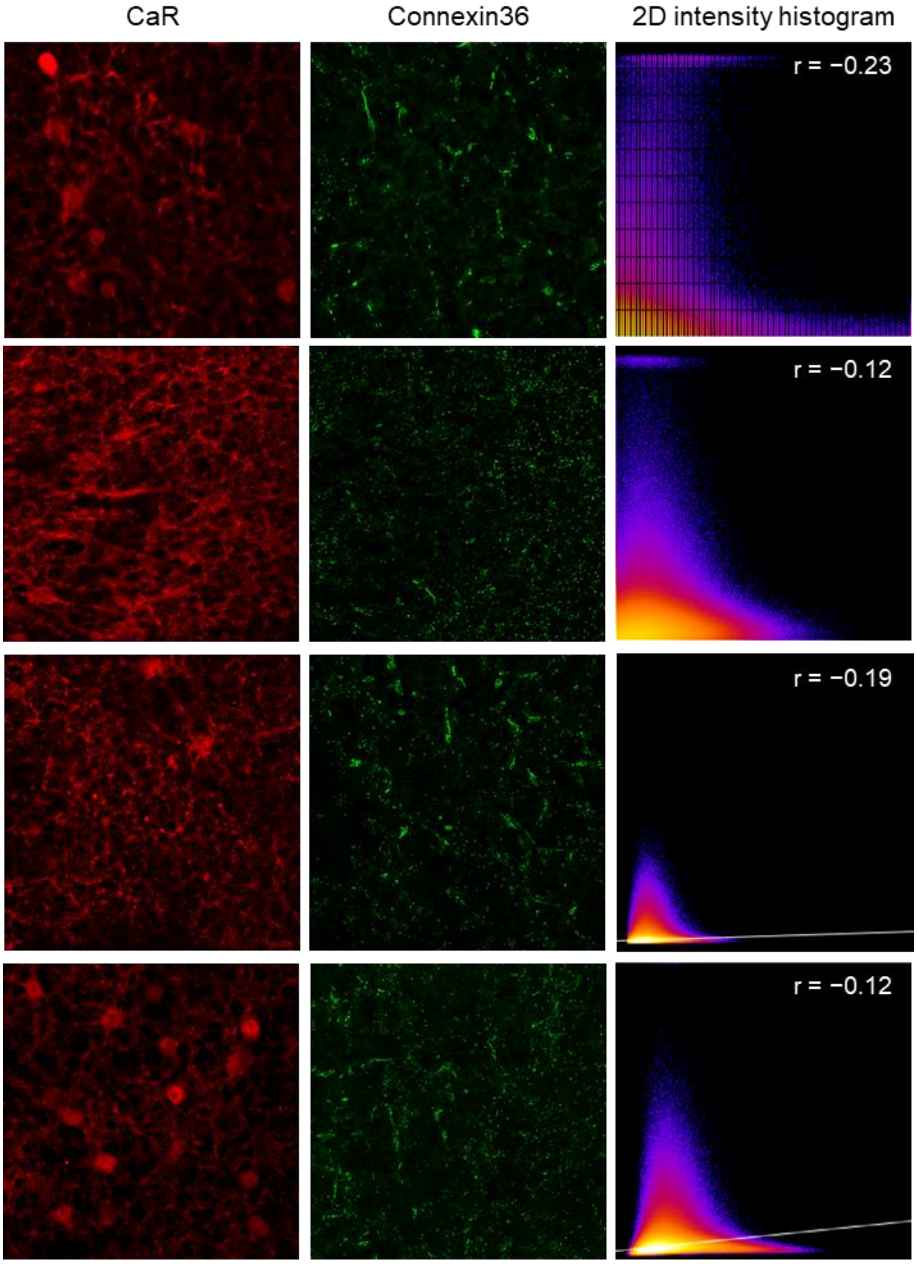

***Supplementary Figure** 1. Colocalization analysis of CaR and Cx36 label in the cat OPL. Images in the columns for CaR and Cx36 are Z-projected from the 10 optical sections on which the colocalization analysis was performed. The 2D intensity histograms show the joint distribution of the CaR (abscissa) and Cx36 (ordinate) label intensities with the relative number of pixels color coded from black (zero) to white (maximum). Pearson's correlation values of above-threshold pixels are shown for each histogram.*

Reference

Schindelin J, White DJ, Kazimiers T, Arena E, Rueden C, Eglinger J, Guizzetti L, Hiner M, Tinevez J-Y (2017) Coloc 2. https://imagej.net/Coloc_2. Accessed 17 February 2019
